# Randomized Controlled Trial to Relieve Pain of Chemotherapy-Induced Peripheral Neuropathy by Magnetic Field: SMILE Study

**DOI:** 10.3390/cancers18040581

**Published:** 2026-02-10

**Authors:** Emi Kubo, Eriko Satomi, Toru Mukohara, Nozomu Fuse, Masashi Wakabayashi, Yuichiro Tsukada, Hiroto Ishiki, Shin Takayama, Yukihide Kanemitsu, Iwao Kishita, Keiko Kobayashi, Chiaki Kurihara, Nami Hirano, Takashi Ikeno, Tomofumi Miura, Akihiro Sato

**Affiliations:** 1 Department of Palliative Medicine, National Cancer Center Hospital East, Kashiwa 277-8577, Japan; ekubo@east.ncc.go.jp; 2 Department of Palliative Medicine, National Cancer Center Hospital, Tsukiji 104-0045, Japan; 3 Department of Medical Oncology, National Cancer Center Hospital East, Kashiwa 277-8577, Japan; tmukohar@east.ncc.go.jp; 4 Clinical Research Support Office, National Cancer Center Hospital East, Kashiwa 277-8577, Japan; nofuse@east.ncc.go.jp (N.F.); mawakaba@east.ncc.go.jp (M.W.); kekobay2@east.ncc.go.jp (K.K.); ckurihar@east.ncc.go.jp (C.K.); nhirano@east.ncc.go.jp (N.H.); tikeno@east.ncc.go.jp (T.I.);; 5 Department of Colorectal Surgery, National Cancer Center Hospital East, Kashiwa 277-8577, Japan; yutsukad@east.ncc.go.jp; 6 Department of Breast Surgery, National Cancer Center Hospital, Tsukiji 104-0045, Japan; stakayam@ncc.go.jp; 7 Department of Colorectal Surgery, National Cancer Center Hospital, Tsukiji 104-0045, Japan; 8 Peace of Mind Co., Ltd., Kumamoto 861-5525, Japan; kishita.2011.1.11@p-mind.co.jp

**Keywords:** CIPN, chemotherapy-induced peripheral neuropathy, paclitaxel, oxaliplatin, magnetic field, breast cancer, colorectal cancer

## Abstract

Background/Objectives: Chemotherapy-induced peripheral neuropathy (CIPN) is a common side effect of taxanes and platinum-based agents, impairing quality of life. Duloxetine is the only drug with proven efficacy, but is often limited by tolerability. AT-04 is a portable alternating magnetic field device that activates descending pain modulatory systems and has shown safety in other pain settings. This investigator-initiated trial evaluated the efficacy and safety of AT-04 for persistent CIPN. Methods: We conducted a randomized, double-blind, sham-controlled, multicenter phase II study. Patients with CIPN lasting ≥12 weeks after chemotherapy and pain NRS ≥ 4 were enrolled. Participants used AT-04 or sham devices twice daily for 12 weeks. The primary endpoint was the change in pain NRS on day 85. Secondary endpoints included tingling NRS, numbness NRS, CIPN20 subscales, and safety. Results: Twenty-eight patients were randomized (14 per arm). No significant difference was observed in pain NRS on day 85 {AT-04: −1.74 ± 0.6 vs. sham device: −1.45 ± 0.6; one-sided *p* = 0.36, effect size (ES) = 0.48}. CIPN20 motor scores improved with AT-04 on days 29 (−9.82 ± 13.8 vs. 0.74 ± 8.7, respectively; *p* = 0.035, ES = 0.92) and 57 (−11.81 ± 10.6 vs. −0.54 ± 6.7, respectively; *p* < 0.01, ES = 1.26). Among patients whose last chemotherapy had ended >1 year earlier (n = 16), motor scores improved from days 29 (−12.43 ± 10.3 vs. 2.38 ± 8.9, respectively; *p* < 0.01, ES = 1.55) to 85 (−11.76 ± 10.1 vs. −0.07 ± 11.5, respectively; *p* = 0.049, ES = 1.08), while sensory/autonomic scales showed trends. No treatment-related adverse events occurred in the AT-04 group. Conclusions: AT-04 did not significantly reduce pain NRS but improved motor function in CIPN patients, especially in those >1 year after chemotherapy. AT-04 may be a promising non-pharmacological option; larger confirmatory trials are required.

## Simple Summary

Chemotherapy-induced peripheral neuropathy (CIPN) is a common side effect of taxanes and platinum-based agents. We conducted a randomized, double-blind, sham-controlled, multicenter phase II study to evaluate the efficacy and safety of AT-04 in patients with persistent CIPN. Participants used devices for 12 weeks. Twenty-eight patients were randomized. For the primary endpoint, no significant difference was observed in pain NRS on day 85 {AT-04: −1.74 ± 0.6 vs. sham: −1.45 ± 0.6; one-sided *p* = 0.36, effect size (ES) = 0.48}. CIPN20 motor scores improved with AT-04 on days 29 (−9.82 ± 13.8 vs. 0.74 ± 8.7, respectively; *p* = 0.035, ES = 0.92) and 57 (−11.81 ± 10.6 vs. −0.54 ± 6.7, respectively; *p* < 0.01, ES = 1.26). Among patients whose last chemotherapy ended >1 year earlier (n = 16), CIPN motor scores improved from day 29 to day 85. No treatment-related adverse events occurred in the AT-04 group. AT-04 may be a promising non-pharmacological option; larger confirmatory trials are required.

## 1. Introduction

Chemotherapy-induced peripheral neuropathy (CIPN) is known as one of the main side effects of regimens that include taxanes, such as paclitaxel and docetaxel, and platinum-based agents, such as oxaliplatin. A meta-analysis of 4179 patients in 31 studies revealed that CIPN prevalence was 68.1% at 1 month and 30% at 6 months after chemotherapy [1]. Furthermore, a meta-analysis encompassing 29 countries demonstrated that the prevalence of painful CIPN was 40.78% (95% CI, 29.08–52.74), while moderate-to-severe CIPN was observed in 49.04% of patients (95% CI, 37.16–60.95) [2].

The main symptoms of CIPN are numbness, tingling, sensory loss, and chronic limb pain. Severely affected patients may become unable to work or live their everyday lives because of movement and autonomic nervous system disorders, and so their quality of life (QOL) can be markedly impaired [3].

The mechanisms of CIPN are not well-understood. However, it has been considered that damage to sensory nerve axons and cell bodies by chemotherapies involves high levels of inflammatory cytokines, low levels of nerve growth factor (NGF), and low levels of intraepidermal nerve fibers [1,4].

Although several randomized controlled trials (RCTs) have been conducted [5,6,7,8,9], only duloxetine has been proven to be effective [10]. Among patients receiving duloxetine, the most frequently reported adverse events were fatigue (7%), insomnia (5%), and nausea (5%); 11% of patients discontinued treatment because of adverse events. Other medications, such as antiepileptic drugs, opioids, and antidepressant drugs (e.g., gabapentin and pregabalin) have not been proven to be effective [5,8]. There are no known standard treatments for CIPN.

This background led to the need to develop a new therapy that has fewer side effects and can be readily used. Recently, non-pharmacological approaches, such as transcutaneous electric nerve stimulation therapy (Scrambler therapy), have been developed. In addition, interventions such as spinal cord stimulation and neurofeedback have been explored for the treatment of CIPN, and although the available evidence remains limited, these approaches have shown some degree of efficacy [11] and the possibilities of nerve repair and pain relief have been reported [12,13].

A portable magnetic field irradiation device (AT-02) that incorporates a combination of mixed alternative magnetic fields at 2 kHz and 83.3 MHz was reportedly effective for painful fibromyalgia patients in a multicenter randomized double-blind trial [14].

AT-04 (Peace of Mind Co., Ltd., Kumamoto, Japan) is the latest model with the same mechanism, is smaller, and has improved versatility. In basic research, the analgesic effects of AT-04 in PSL model rats involve both the endogenous pain modulation systems, including the descending pain modulatory system, such as the serotonin and noradrenaline systems, and the opioid analgesic system [15]. Clinically, its effectiveness and safety have been demonstrated in patients with cancer pain [16] and pain caused by arteriovenous fistula puncture on conducting hemodialysis [17].

The mechanism of AT-04 involves the descending pain modulatory system, like duloxetine, and so it is considered that AT-04 may be effective against CIPN. In this investigator-initiated clinical trial, we aimed to evaluate the efficacy and safety of AT-04 for CIPN patients.

## 2. Materials and Methods

### 2.1. Trial Design and Participants

We conducted a double-blind, sham device-controlled, phase II, randomized trial in Japan.

It included patients who developed CIPN during perioperative chemotherapy and had residual pain of numeric rating scale (NRS) 4 or higher at least 12 weeks after the completion of chemotherapy, paclitaxel for breast cancer patients, or oxaliplatin for colorectal cancer patients. Participants were also required to: be aged 18 to 80 years old, have no history of receiving treatment for CIPN, not have received stable regular treatments (including drug prescription, dosage, administration) for 14 days prior to providing informed consent, and show stable CIPN symptom severity for 14 days also prior to informed consent. Eligible patients had an Eastern Cooperative Oncology Group (ECOG) performance status <3 and a life expectancy of ≥12 weeks.

Exclusion criteria included: comorbidities presenting with peripheral neuropathy, edema of the limbs, trauma or surgery to the fingers or toes, receiving major surgery within 4 weeks prior to informed consent, using life-supporting medical electrical equipment, exhibiting psychiatric disorders, and administered medications for other diseases that are effective against peripheral neuropathy.

The study (jRCT2032220295) was conducted according to the Declaration of Helsinki and approved by the Institutional Review Board of the National Cancer Center. All patients provided written informed consent.

### 2.2. Study Procedures

Eligible patients were randomly assigned in a 1:1 ratio to sham device or AT-04 groups using the minimization method. Randomization was stratified by a drug that caused CIPN (paclitaxel or oxaliplatin) and the previous use of medications for CIPN (current use also included).

The four pads of the investigational device were applied to four points in both axillae and the groin area for 30 min per session, at least twice a day (maximum of 2 h), for 12 weeks. Patients and site staff were unaware of treatment group assignments. AT-04 and sham devices were identical in appearance and sound, and participants did not perceive any sensation with either device.

### 2.3. Study Endpoints

The primary endpoint was the change in pain NRS on day 85. Secondary outcome endpoint measures were: tingling NRS, numbness NRS, the European Organization for Research and Treatment of Cancer (EORTC) Quality of Life Questionnaire Core 30 (C30), and chemotherapy-induced peripheral neuropathy module (CIPN20) on days 15, 29, 57, 85, and 113, safety, and tolerability.

CIPN-20 is a 20-item questionnaire that enables the evaluation of subscales for sensory, motor, and autonomic nerves [18]. Safety was evaluated using CTCAE ver. 5.0 by a physician.

### 2.4. Statistical Analysis

The planned sample size was set at 28 to detect a 0.75-point decrease in pain NRS on day 85, assuming a standard deviation of 1.0, a one-sided alpha level of 0.15, and 80% power. In exploratory randomized phase II screening trials, less stringent type I error rates have been proposed to balance feasibility and the risk of missing potentially active treatments. Rubinstein suggested that type I and type II error rates in the range of 10–20% may be appropriate in such designs [19]. Based on this framework and feasibility considerations, a one-sided alpha level of 0.15 was pre-specified for the primary endpoint. Similar one-sided significance levels (α = 0.15) have been adopted in selected exploratory randomized phase II trials aimed at signal detection, such as those reported by Yoon [20] and Omuro [21].

The primary analysis of the change in pain NRS on day 85 was performed using analysis of covariance (ANCOVA), adjusting for the stratification factors used in randomization. For secondary and other exploratory analyses, including pain NRS, tingling NRS, numbness NRS, and EORTC QLQ-CIPN20, group comparisons were conducted using two-sided tests with a conventional significance level of 0.05. In figures, asterisks (*) indicate results with two-sided *p*-values < 0.05, whereas results meeting the prespecified one-sided alpha level of 0.15 for the primary endpoint are described in the text. Subgroup analyses were conducted according to the neurotoxic agent (oxaliplatin vs. taxane) and time since completion of chemotherapy (<1 year vs. ≥1 year) and were considered hypothesis-generating. All statistical analyses were performed using SAS version 9.4 (Cary, NC, USA).

## 3. Results

**Figure 1:**
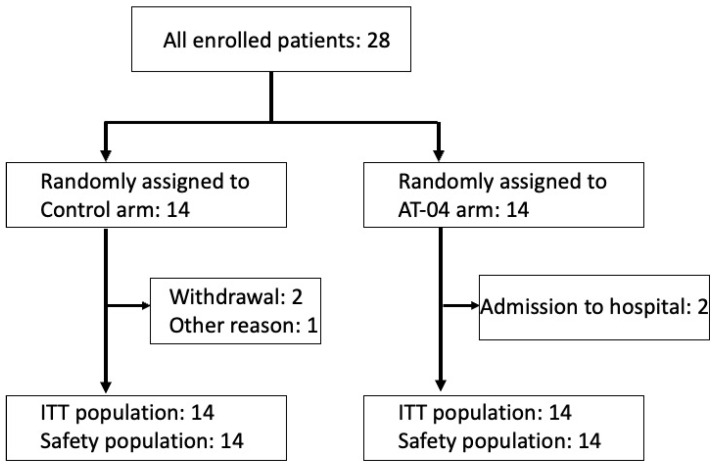
CONSRT diagram.

Between September 2022 and July 2023, of the 28 patients registered for the study, 14 were allocated to AT-04 and sham device groups, respectively (Figure 1).

Patient characteristics are described in Table 1.

The median ages were 66 years (IQR: 58–69) in the sham device group and 59.5 years (55–69) in the AT-04 group. Eleven (78.6%) and 10 (71.4%) were women, respectively. The median time after neurotoxic agents was 1207.5 and 560.5 days, respectively, but no differences were observed in other characteristics, such as histories of diabetes or medication for CIPN.

Also, there was no difference in pain NRS (Table 2).

### 3.1. Pain NRS

**Figure 2:**
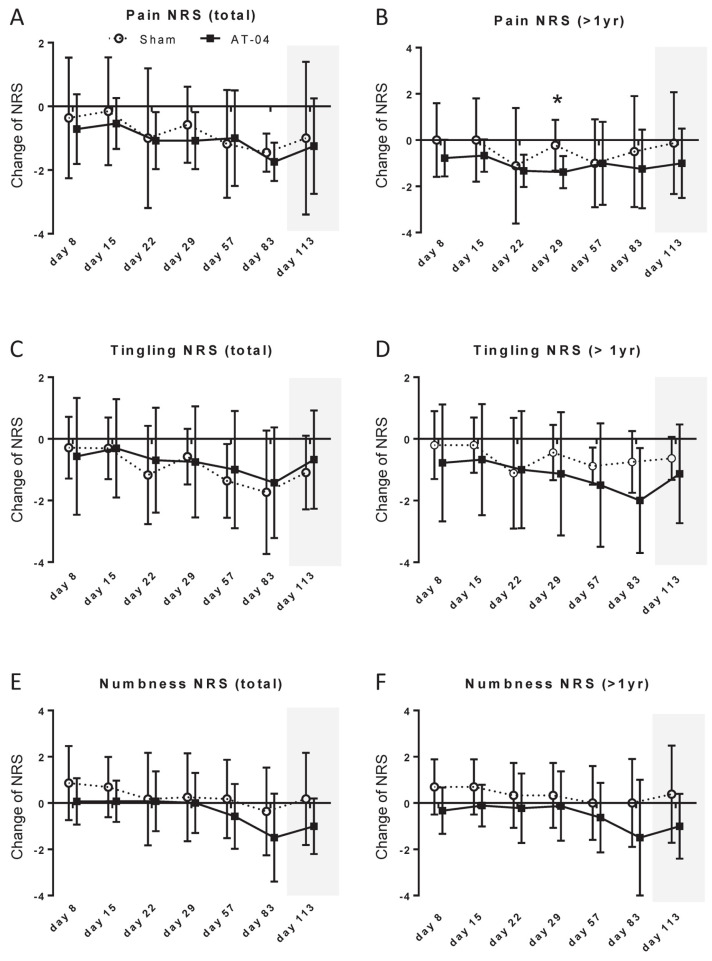
Changes in pain/tingling/numbness NRS. (**A**) pain NRS (total), (**B**) pain NRS (>1 year), (**C**) tingling NRS (total), (**D**) tingling NRS (>1 year), (**E**) numbness NRS (total), (**F**) numbness NRS (>1 year). *, *p* < 0.05.

The mean (SD) change in pain NRS from Day 1 was −1.45 (0.6) in the sham device group and −1.74 (0.6) in the AT-04 group, and the difference in the change (95% CI, effect size (ES)) was −0.29 (−1.95 to 1.37, *p* = 0.48) (Table S1). In a one-sided test of the difference in changes in pain NRS using analysis of covariance with allocation of adjustment factors (neurotoxic agents and history of medication for CIPN) as covariates, the result was *p* = 0.35916. This did not meet the significance level of less than 15%. The change in pain NRS for all patients is shown in Figure 2A, and this change for patients whose last chemotherapy occurred more than one year prior is presented in Figure 2B. The difference in the change on Day 29 for patients whose last chemotherapy occurred more than one year prior was −1.15 (−2.13~−0.17, *p* = 0.024, ES = 1.24) (Table S1).

### 3.2. Tingling NRS

The change in tingling NRS for all patients is shown in Figure 2C, and this change for patients whose last chemotherapy occurred more than one year prior is presented in Figure 2D. In patients whose last chemotherapy occurred more than one year prior, a trend toward a greater change at Day 85 was observed (difference: −1.25; 95% CI, −2.75 to 0.25; *p* = 0.096; ES = 0.90) (Table S1).

### 3.3. Numbness NRS

The change in numbness NRS for all patients is shown in Figure 2E, and this change for patients whose last chemotherapy occurred more than one year prior is presented in Figure 2F. In all patients, a trend toward a greater change was observed at Day 113 (difference: −1.18; 95% CI, −2.59 to 0.23; *p* = 0.097; ES = 0.72) (Table S1). In patients whose last chemotherapy occurred more than one year prior, a trend toward a greater change was observed at Day 8 (difference: −1.03; 95% CI, −2.09 ~ 0.02, *p* = 0.054, ES = 0.93) on Day 8 (Table S1).

### 3.4. General Health-Related Quality of Life

The change in the C-30 summary score for all patients and this change for patients whose last chemotherapy occurred more than one year prior is presented in Table S1. In patients whose last chemotherapy occurred more than one year prior, a trend toward a greater change was observed at Day 57 (difference: 6.02; 95% CI, −1.22 ~ 13.25, *p* = 0.096, ES = 0.93) and Day 85 (difference: 7.43; 95% CI, −1.06 ~ 15.92, *p* = 0.081, ES = 0.94) (Table S1).

### 3.5. CIPN-20 Sensory Scale

**Figure 3:**
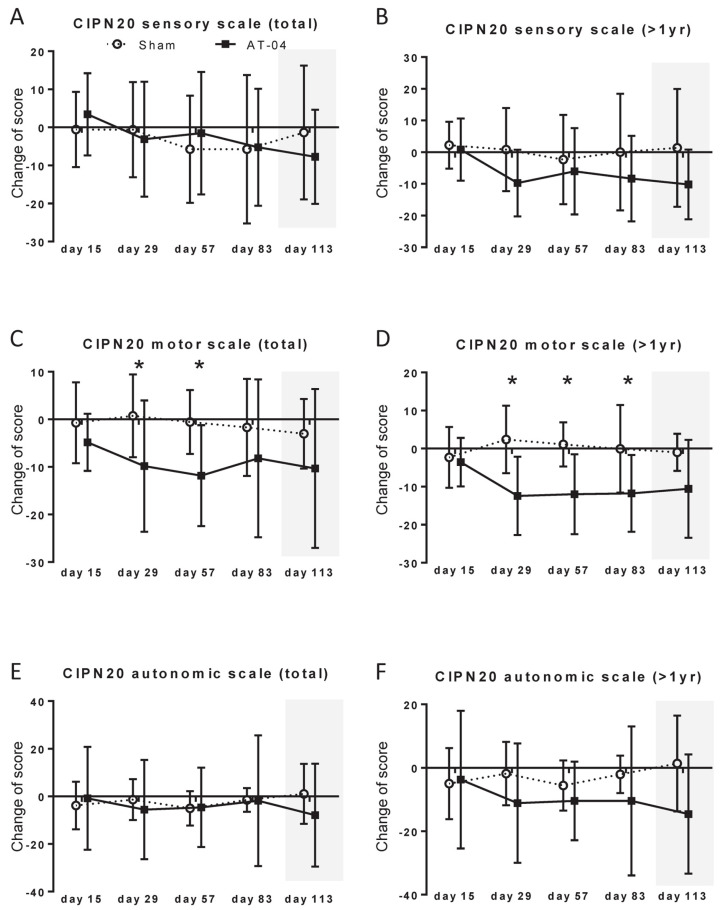
Changes in EORTC QLQ-CIPN20. (**A**) CIPN20 sensory scale (total), (**B**) CIPN20 sensory scale (>1 year), (**C**) CIPN20 motor scale (total), (**D**) CIPN20 motor scale (>1 year), (**E**) CIPN20 autonomic scale (total), (**F**) CIPN20 autonomic scale (>1 year). *, *p* < 0.05.

The change in the CIPN-20 sensory scale for all patients is shown in Figure 3A, and this change for patients whose last chemotherapy occurred more than one year prior is presented in Figure 3B. No significant difference was observed in any of the patients. In patients whose last chemotherapy occurred more than one year prior, a trend toward a greater change was observed at Day 29 (difference:10.55; 95% CI, −22.89 ~ 1.80, *p* = 0.089, ES = 0.88) (Table S1).

### 3.6. CIPN-20 Motor Scale

The change in the CIPN-20 motor scale for all patients is shown in Figure 3C, and this change for patients whose last chemotherapy occurred more than one year prior is presented in Figure 3D. The difference in the change for all patients was −10.57 (−20.34~−0.79, *p* = 0.035, ES = 0.92) on Day 29 and −11.26 (−19.02~−3.50, *p* = 0.007, ES = 1.26) on Day 57 (Table S1). In patients whose last chemotherapy occurred more than one year prior, this difference for patients whose last chemotherapy occurred more than one year prior was −14.81 (−24.70~−4.91, *p* = 0.006, ES = 1.55) on Day 29, −13.10 (−22.19~−4.00, *p* = 0.008, ES = 1.54) on Day 57,−11.68 (−23.28~−0.09, *p* = 0.049, ES = 1.08) on Day 85, and a trend toward a greater change was observed at Day 113 (difference: −9.60; 95% CI, −20.09~0.90, *p* = 0.070, ES = 0.98) (Table S1).

### 3.7. CIPN-20 Autonomic Scale

The change in the CIPN-20 autonomic scale for all patients is shown in Figure 3E, and this change for patients whose last chemotherapy occurred more than one year prior is presented in Figure 3F. No significant difference was observed in any of the patients (Table S1). In patients whose last chemotherapy occurred more than one year prior, a trend toward a greater change was observed at Day 113 (difference: −15.97; 95% CI, −34.22 ~ 2.28, *p* = 0.082, ES = 0.94) (Table S1).

### 3.8. Treatment-Related Adverse Events (trAEs)

Two trAEs occurred in 2 patients of the sham device group (Table 3). They were Grade 1 dizziness and Grade 3 peripheral sensory neuropathy. No trAE occurred in the AT-04 group.

## 4. Discussion

This study was a clinical trial to explore the efficacy and safety of the alternating magnetic field therapy device, AT-04, for CIPN patients whose symptoms persisted for longer than three months after regimens that included neurotoxic agents.

No significant difference was observed in any of the patients regarding pain NRS, tingling NRS, or numbness NRS, and ad hoc analyses revealed moderate to strong effect sizes. Improvements in the CIPN-20 motor scale were observed on Days 29 and 57.

In exploratory subgroup analyses of patients whose last chemotherapy was received more than one year prior, differences in tingling NRS and numbness NRS were observed between groups. Ad hoc analyses suggested relatively large effect sizes. Improvements in the CIPN-20 motor scale were observed from Day 29 onwards.

The important finding was that AT-04 showed a moderate effect size, which was of a similar magnitude to that reported for duloxetine, although the primary endpoint of pain NRS did not reach statistical significance. Duloxetine remains the only agent with established efficacy for CIPN; in the CALGB 170601 trial, a significant improvement in mean pain scores was reported, with an effect size of 0.51 [10]. The effect size observed for AT-04 in the present study was within a comparable range, providing contextual information regarding the magnitude of the observed effects rather than evidence of equivalent efficacy.

In exploratory subgroup analyses of patients whose last chemotherapy occurred more than one year prior, improvements were observed not only in pain NRS but also in tingling NRS and numbness NRS, with effect sizes in the moderate to large range. These findings should be interpreted cautiously, as they were derived from secondary and ad hoc analyses; however, they may suggest that the clinical manifestations of CIPN and their responsiveness to intervention can change over time.

The acute phase of CIPN is primarily caused by peripheral mechanisms such as axonal degeneration, microtubule dysfunction, mitochondrial dysfunction, and inflammatory cytokines [22]. A paclitaxel-induced neuropathy model showed that persistent activation of spinal astrocytes is involved in pain, suggesting that central glia cells are involved in the pathogenesis of chronic CIPN [23]. Therefore, it is considered that central sensitization via glial activation and neuroinflammation is essential to maintain pain in the chronic phase, although CIPN is primarily a peripheral disorder in the acute phase [24]. Clinically, CIPN patients with pain exhibit enhanced temporal summation and reduced conditioned pain modulation (CPM), indicating the presence of central sensitization and impaired descending inhibitory control [25]. Furthermore, in the study by Boland using fMRI to investigate central pain processing in patients with chronic CIPN, overactivation of the insular cortex and somatosensory cortex (S1/S2) and decreased activity of the right superior frontal gyrus were observed in patients with CIPN, suggesting sensitization of pain-related centers and dysfunction of inhibitory networks [26].

Integrating these clinical and basic research findings supports the idea that in the chronic phase of CIPN, central sensitization and failure of descending inhibition, as well as neuroinflammation caused by spinal glial activation, are involved in the persistence of symptoms. In this study, the observation that AT-04 appeared to be more effective in patients whose last chemotherapy occurred more than one year prior may be consistent with a neuromodulatory effect acting on these central mechanisms. However, this interpretation remains speculative and should be considered hypothesis-generating.

In fact, as shown by Kohno using the partial sciatic nerve ligation (PSL) model, AT-04, or alternating magnetic field therapy, increased spinal cord serotonin and noradrenaline concentrations, and this effect was suppressed by approximately 50% with 5-HT and α_2_ receptor antagonists, and by approximately 60% with naloxone. Therefore, it was suggested that the mechanism of action of AT-04 involves activation of the descending inhibitory and endogenous opioid systems [15].

Scrambler therapy has also been reported to be effective against CIPN; its mechanism has not been fully verified neuroscientifically but involves the concept of “replacement of pain signals” [10]. In this respect, AT-04 is a non-pharmacological intervention with an evidence-based mechanism of action that can be positioned as “non-pharmacological SNRI”. In addition, this device is small, portable, and can be used by patients themselves, whereas scrambler therapy can only be administered by trained personnel. Other approaches, such as neuromodulation devices and neurofeedback, currently lack robust evidence and warrant further investigation and development [11,27,28].

Another important finding was an association between AT-04 treatment and early changes in the CIPN-20 motor score. Most RCTs of drug therapy against CIPN selected pain NRS as the primary endpoint, and few reports primarily showed improvement in the motor function. In fact, the focus of RCTs involving duloxetine has been on improving pain, and no clear evidence has been presented regarding improvement in the motor function [10]. Gabapentin [5], pregabalin, and vitamin B12 [8] all have limited efficacy regarding the reduction in pain, and no improvement in the motor function has been confirmed using them. It is known that motor disorders in CIPN patients are caused by a combination of abnormal peripheral sensory input, proprioceptive dysfunction, and defensive motor inhibition associated with pain [7]. Numbness is considered to reflect small fiber neuropathy. This is demonstrated by the reduced IENFD observed in skin biopsies from patients with CIPN [29,30]. However, the correlation between symptom severity and IENFD is inconsistent across medications or time periods [31].

Furthermore, tingling suggests the involvement of central input amplification and reduced descending inhibition [25], as evidenced by enhanced temporal summation in quantitative sensory testing, diminished conditioned pain modulation, excessive activation in the insular cortex and S1/S2, and reduced activity in the right superior frontal gyrus during thermal pain fMRI [26]. Therefore, the observed improvements in tingling and numbness with AT-04 in this study may be associated with subsequent changes in motor function. One possible explanation is that modulation of sensory abnormalities, potentially through engagement of descending inhibitory pathways and altered processing of residual sensory input, may influence motor performance. Improvement in motor function is clinically meaningful for patients with CIPN; however, the underlying mechanisms remain speculative and warrant further investigation. CIPN patients have been shown to exhibit slower walking speeds, shorter stride lengths, and higher scores on the Timed Up and Go test [32]. Furthermore, such patients experience more severe muscle weakness and balance disorders, which can increase the risk of falls [3]. The observed improvements in CIPN-20 motor scores may indicate a potential association between AT-04 treatment and functional aspects of CIPN. While motor function is closely related to activities of daily living and quality of life in cancer survivors, the present findings are exploratory and do not allow conclusions regarding direct effects on these outcomes.

Limitations: This study had several limitations. First, this study was designed as an exploratory investigation. Accordingly, in light of the inherent limitations of the exploratory design and feasibility constraints, a one-sided significance level of 15% was prespecified and applied strictly for exploratory purposes. In addition, to provide complementary context for the observed effects, effect sizes were calculated, and the results were compared with those reported in randomized controlled trials of duloxetine. Second, the primary endpoint was set as pain NRS. Because pain is multifactorial and markedly influenced by psychosocial factors, a more sensitive evaluation may have been possible by combining multiple indices, such as worst pain NRS and BPI, rather than single pain NRS. Third, biomarkers such as NGF were not measured, so it was not possible to directly verify the mechanism of action. Fourth, the experimental model used by Kohno is the partial sciatic nerve ligation (PSL) model, which is not a CIPN-specific model. Therefore, extrapolating the mechanism of AT-04 directly to CIPN requires careful interpretation. Despite these limitations, AT-04 demonstrated an efficacy equivalent to duloxetine and improved CsIPN-20 motor scores, tingling NRS, and numbness NRS. Consequently, we consider the efficacy of AT-04 for CIPN patients to be promising.

## 5. Conclusions

In this exploratory trial, the AT-04 alternating magnetic field therapy device was unable to demonstrate significant improvement in pain NRS scores in CIPN patients whose symptoms persisted for more than three months after the discontinuation of neurotoxic agents. However, it showed a moderate effect on pain and numbness, and the effect was particularly promising in patients whose last chemotherapy occurred more than one year prior. Furthermore, the observed improvement in the CIPN-20 motor score suggests a finding that has not been previously reported in medication-based treatment trials for CIPN. AT-04 possesses central mechanisms involving activation of the descending inhibitory and endogenous opioid systems and may contribute to CIPN treatment as a non-pharmacological therapeutic option. In the future, further clarification of its efficacy and mechanism will be warranted through large-scale validation studies and biomarker analyses.

## Figures and Tables

**Table 1:** Patient characteristics.

Characteristic	Sham (n = 14) n (%)	AT-04 (n = 14) n (%)	*p*-Value	Total (n = 28) n (%)
Age(y), Median (IQR)	66 (58–69)	59.5 (55–69)	0.597	62 (56–69)
Sex				
Female	11 (78.6)	10 (71.4)	0.663	21 (75.0)
Cancer				
Colorectal	5 (35.7)	5 (35.7)	1	10 (35.7)
Breast	9 (64.3)	9 (64.3)		18 (64.3)
ECOG-PS				
0	10 (71.4)	10 (71.4)	1	20 (71.4)
1	4 (28.6)	4 (28.6)		8 (28.6)
Chemotherapy				
Paclitaxel	9 (64.3)	9 (64.3)	1	18 (64.3)
Oxaliplatin	5 (35.7)	5 (35.7)		10 (35.7)
Period after chemotherapy				
More than 1 year	10 (71.4)	9 (64.3)	0.686	19 (67.9)
Previous use of medication for CIPN				
Yes	10 (71.4)	11 (78.6)	0.663	21 (75.0)
Diabetes				
Yes	2 (14.3)	2 (14.3)	1	4 (14.3)
BMI				
≥25	6 (42.9)	4 (28.6)	0.430	10 (35.7)

IQR, interquartile range; ECOG-PS, Eastern Cooperative Oncology Group Performance Status; CIPN, chemotherapy-induced peripheral neuropathy; BMI, body mass index.

**Table 2:** Baseline scores.

Characteristic	Sham (n = 14) Mean (SD)	AT-04 (n = 14) Mean (SD)	*p*-Value
Pain NRS	5.2 (1.5)	5.8 (1.7)	0.354
Tingling NRS	5.7 (1.6)	5.9 (1.2)	0.697
Numbness NRS	4.7 (2.3)	5.9 (2.0)	0.172
C-30 Summary Score	83.8 (8.6)	77.5 (10.6)	0.097
CIPN-20 sensory scale	27.5 (12.7)	31.7 (14.9)	0.427
CIPN-20 motor scale	22.9 (17.6)	35.2 (20.0)	0.094
CIPN-20 autonomic scale	21.0 (18.4)	19.8 (21.3)	0.876

SD, standard deviation; NRS, numeric rating scale; CIPN, chemotherapy-induced peripheral neuropathy.

**Table 3:** Treatment-related AE.

	Sham (n = 14)	AT-04 (n = 14)	Total	
Grade 1	1	0	1	Dizziness
Grade 2	0	0	0	
Grade 3	1	0	1	Peripheral sensory neuropathy
Grade 4	0	0	0	
Grade 5	0	0	0	
Total	2	0	2	
Grade 3≦	1	0	1	

## Data Availability

In accordance with the protocol, the data will be available for researchers who have submitted a research proposal and statistical analysis plan and obtained approval from the clinical trial coordination committee, the clinical trial device provider, and the IRB.

## Supplementary Materials

The following supporting information can be downloaded at https://www.mdpi.com/article/10.3390/cancers18040581/s1, Table S1: Comparison between all patients and patients whose last chemotherapy occurred more than one year prior.
